# Supplementary material for: Modulation of polypeptide conformation through donor–acceptor transformation of side-chain hydrogen bonding ligands
Source: Nat Commun. 2017 Jul 21;8:92. doi: 10.1038/s41467-017-00079-5 (PMC5522480; doi:10.1038/s41467-017-00079-5)
Supplement: Supplementary file 1 — Supplementary Information [file 41467_2017_79_MOESM1_ESM.pdf]

File Name: Supplementary Information
Description: Supplementary Figures, Supplementary Table, Supplementary Discussion, Supplementary Methods and Supplementary References

File Name: Supplementary Movie 1
Description: . Movie of the 100 ns MD simulation trajectory of a P1 polypeptide 10- mer under neutral pH conditions where the side chains contain 1,2,3-triazole groups (P1- triazole). Frames are rendered every 100 ps.

File Name: Supplementary Movie 2
Description: . Movie of the 100 ns MD simulation trajectory of a P1 polypeptide 10- mer under acidic pH conditions where the N3 in the 1,2,3-triazole protonates to form a triazolium group (P1- triazolium). Frames are rendered every 100 ps.

File Name: Peer Review File
Description:

Supplementary Discussion

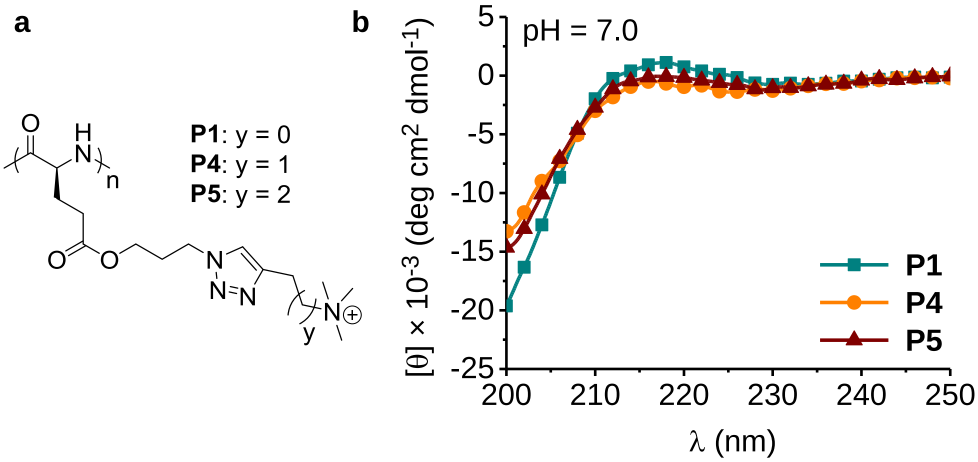

Supplementary Figure 1. Secondary structure characterization of **P4** and **P5**. **a**, Chemical structure of **P4** and **P5**. **b**, CD spectra of **P4** and **P5** at pH 7.0.

In contrast to the α-helical conformation of **P3** (Fig. 2e), **P4** and **P5** (extended distances between ammonium and triazole compared with **P1**) exhibited a typical random coil conformation at pH 7.0 from CD. This result suggests that the disruptive effect comes from side-chain triazoles rather than the side-chain ammonium cations.

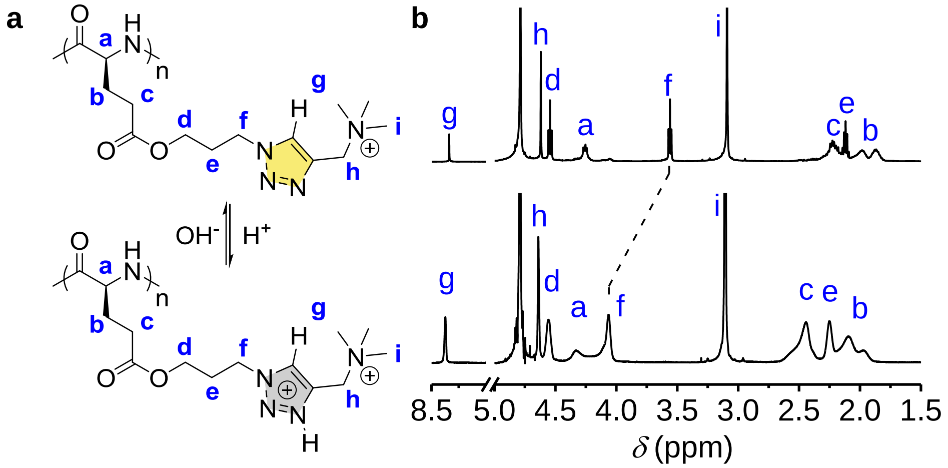

Supplementary Figure 2. Chemical structures (**a**) and ^{1}H NMR spectra (**b**) of **P1** in D_2O under basic (top, pH ~ 12) and acidic (bottom, pH ~ 2) conditions.

We are able to obtain two pieces of information about the pH-sensitive conformation change of **P1** based on ^{1}H NMR. A significant downfield shift of the methylene protons adjacent to triazole C1 (proton *f*) was observed upon the addition of acid, indicating the protonation of triazoles; The sharp peaks for all backbone protons at high pH (proton *a*-*c*) became weaker and broader at low pH because of the side-chain shielding upon polypeptide folding[26].

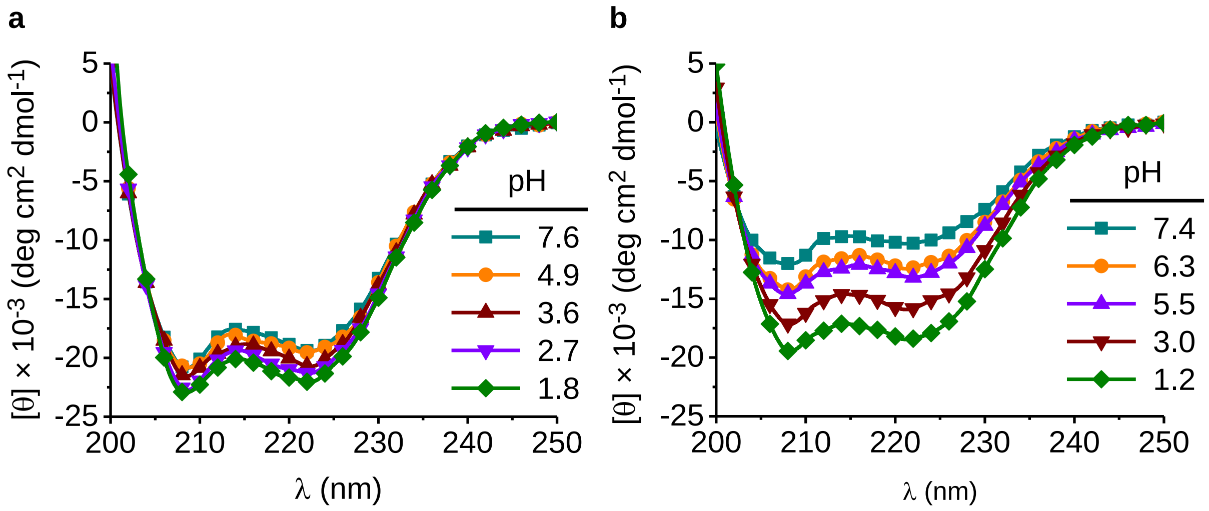

Supplementary Figure 3. CD spectra of **P2** (**a**) and **P3** (**b**) upon stepwise addition of concentrated HCl.

In contrast to the pH-induced conformation change of **P1** (Fig. 3b), **P2** adopted a stable α-helical conformation over a wide pH range of 1-11. This result confirms the pH-induced change in polypeptide conformation originates from the side-chain triazoles. The conformation of control polypeptide **P3**, on the other hand, is pH-dependent due to the side-chain triazoles. Although the longer distance between triazole and backbone weakens the disrupting effect of triazole in **P3**, it cannot completely block the disruption. This also explains why **P3** has a much lower helicity than **P2** at pH 7.0 (Figure 2e).

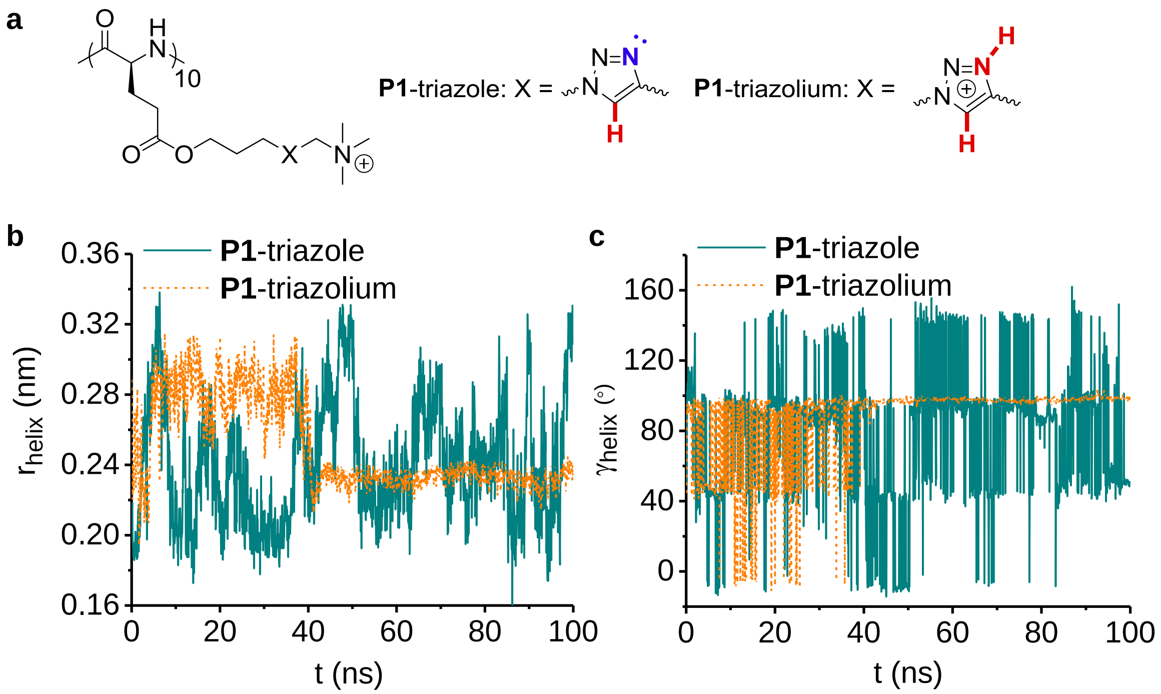

Supplementary Figure 4. MD simulations of **P1** (10-mer) in **P1**-triazole and **P1**-triazolium forms. Polypeptides are initialized as extended random coils and simulated for 100 ns. **a**, Chemical structure of **P1**-triazole and **P1**-triazolium. **b**, The effective helix radius r_{helix} calculated from the mean radial distance of the backbone Cα atoms in a two-dimensional projection orthogonal to a best fit of the polypeptide backbone to an ideal helix ($r_{\text{helix}}^{\text{ideal}}$ = 0.23 nm). **c**, The twist angle γ_{helix} between successive residues projected along the best fit of the polypeptide backbone to an ideal helix ($\gamma_{\text{helix}}^{\text{ideal}}$ = 100 °).

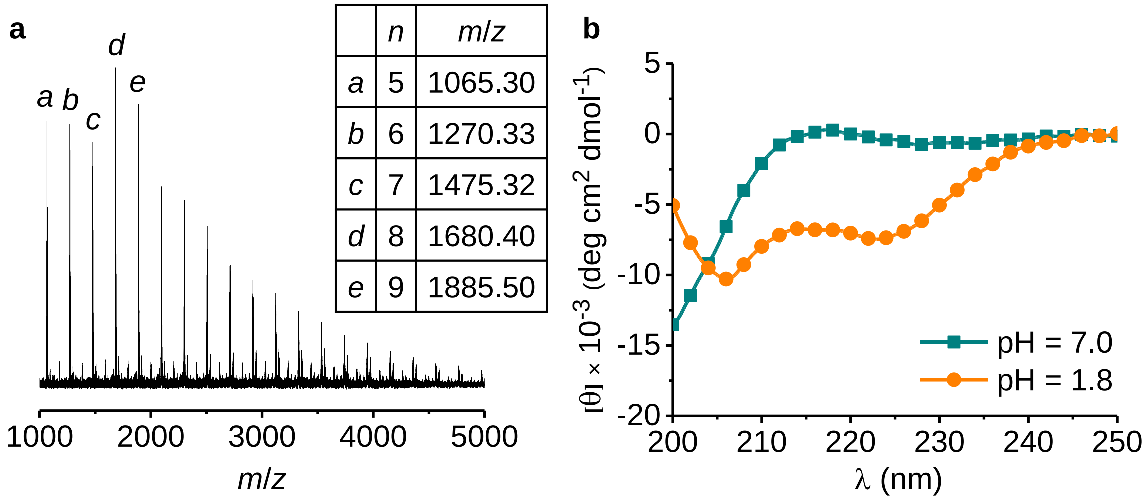

Supplementary Figure 5. Secondary structure characterization of **P1** (10-mer). **a**, MALDI-TOF spectrum of chlorine-functionalized **P1** precursor (PCPLG, 10-mer). **b**, CD spectra of **P1** (10-mer) under acidic and basic conditions.

The chlorine-functionalized **P1** precursor (10-mer) is too short to show a good GPC trace. We therefore use MALDI-TOF mass spectrometry instead to characterize this polypeptide precursor. All peaks are in excellent agreement with the calculated *m*/*z* value ((41.00 + 205.05 *n*), which corresponds to $[M + Na]^+$).

P1 (10-mer) has similar pH-sensitive conformation change compared with **P1** (50-mer). The mean residue molar ellipticity at 222 nm predicted from simulation trajectories agrees very well with the experimental CD results of **P1** (10-mer).

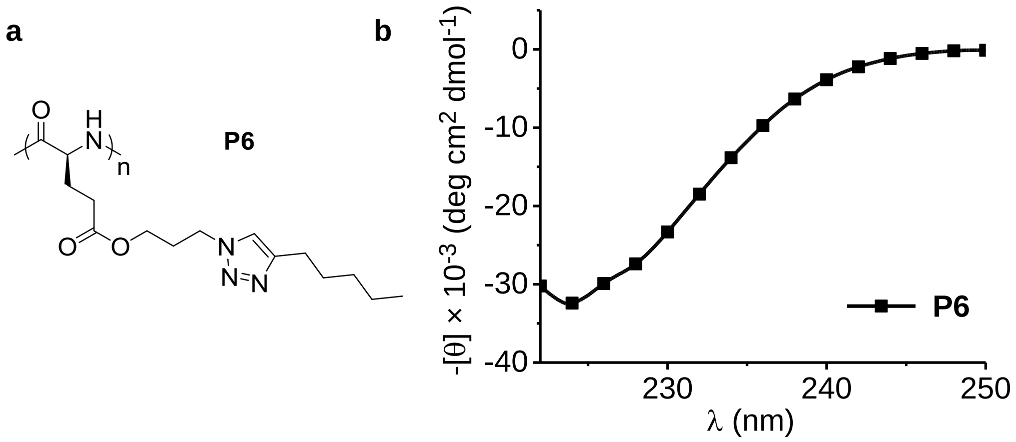

Supplementary Figure 6. Secondary structure characterization of **P6**. **a**, Chemical structure of **P6**. **b**, CD spectrum of **P6** in $CHCl_3$.

P6, an organic solvent soluble triazole polypeptides, still adopts typical α-helical conformation in $CHCl_3$ even the side-chain triazole stays in its non-protonated form. $CHCl_3$ as the solvent molecule cannot stabilize the backbone carbonyl and N-H groups. **P6** therefore tends to stay as α-helix in non-aqueous solvent, with all its backbone amides H-bonded with each other.

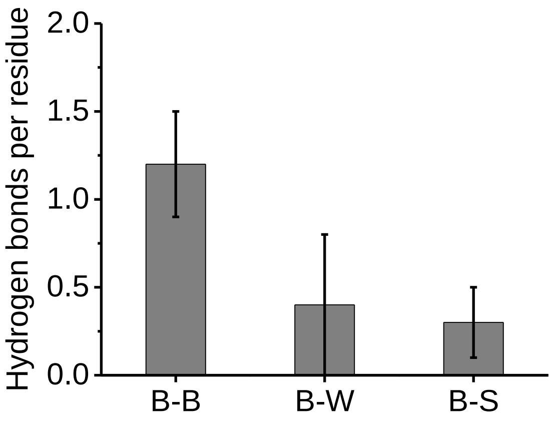

Supplementary Figure 7. Analysis of H-bonding partners for the central residues (Residue 5 and Residue 6) of **P1**-triazolium within the polypeptide backbone (B-B), between the backbone and water (B-W), and between the backbone and side-chain triazolium (B-S). Error bars are standard deviations about the mean of the distributions of H-bonds per residue. Each distribution contains 1000 data points.

Due to the short backbone length of the 10-mer peptides studied by MD simulation, the dangling peptide bond at the chain terminal accounts for a great percentage of the whole backbone peptide groups. This will affect the analysis of the H-bonding partners for **P1**-triazolium (Fig. 4c), since the four dangling carbonyl/N-H groups at each end are unable to form H-bonds with other backbone peptides even in an α-helical conformation. We therefore took the central residue (Residue 5 and Residue 6) of **P1**-triazolium and analyzed their H-bonding partners, which showed an even higher number of intramolecular backbone-backbone H-bonds.

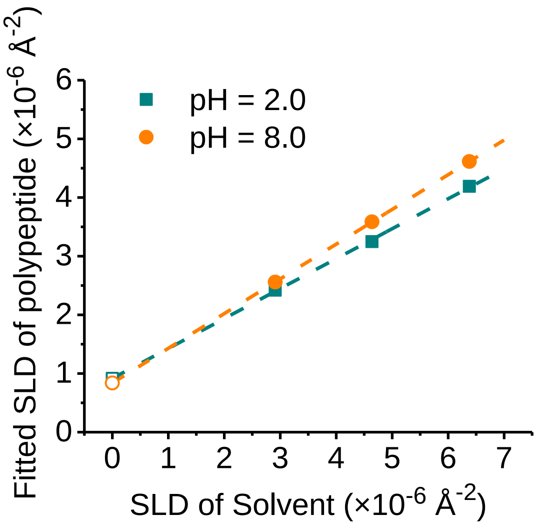

Supplementary Figure 8. Confirmation of $SLD_{h\text{-}pep}$ with the intercepts obtained from fitting in Fig. 4f. The intercepts in both cases fitted with the linear fitting curve, indicating the best-fitting $SLD_{h\text{-}pep}$ and obtained φ_{sol} are reasonable.

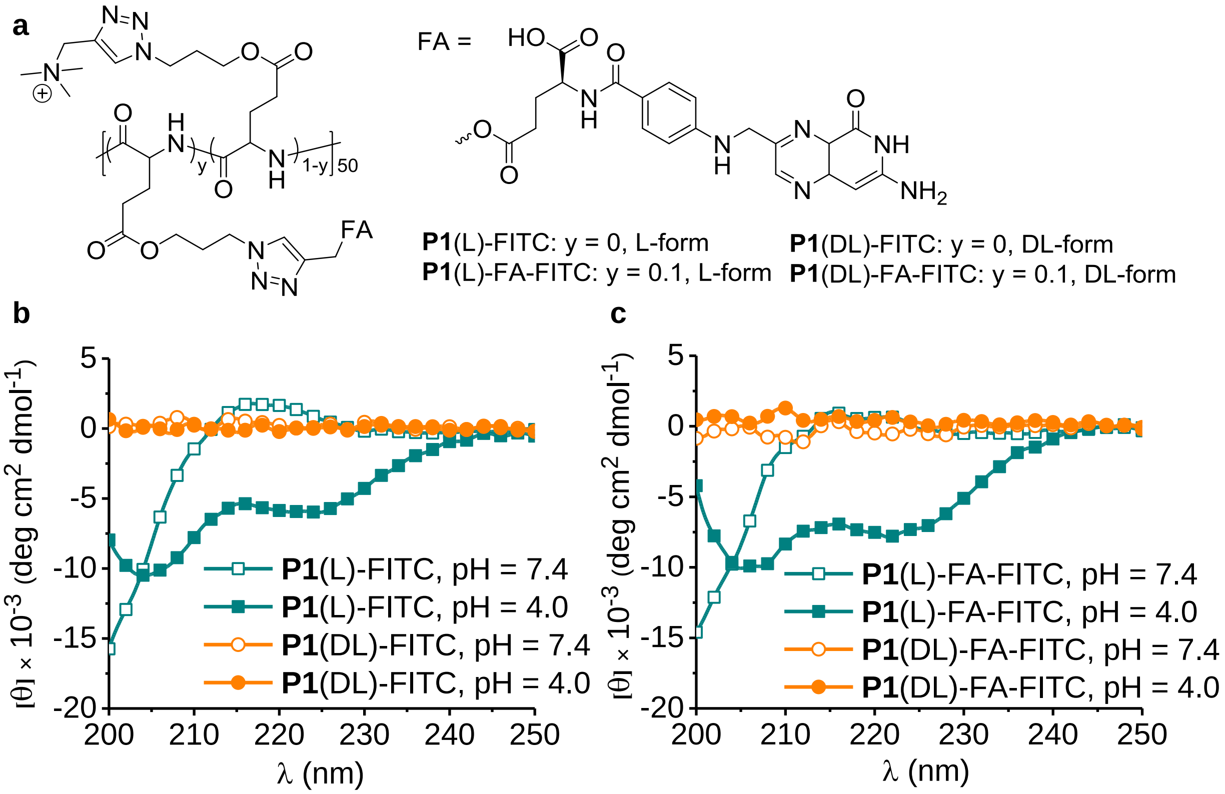

Supplementary Figure 9. Secondary structure characterization of FITC-labeled and folic acid (FA)-decorated triazole polypeptides. **a**, Chemical structure of polypeptides. **b**, **c**, CD spectra of polypeptides at pH = 7.4 (open) and at pH = 4.0 (solid).

The DL form of polypeptides (**P1**(DL)-FITC and **P1**(DL)-FA-FITC) was used as a control, where no Cotton effect was observed (flat curve with ellipticity around 0 at wavelength 200-250 nm). The introduction of fluorescent dye FITC and the targeting agent folic acid (FA) showed negligible effect on pH-dependent helix-coil transition behavior of **P1**.

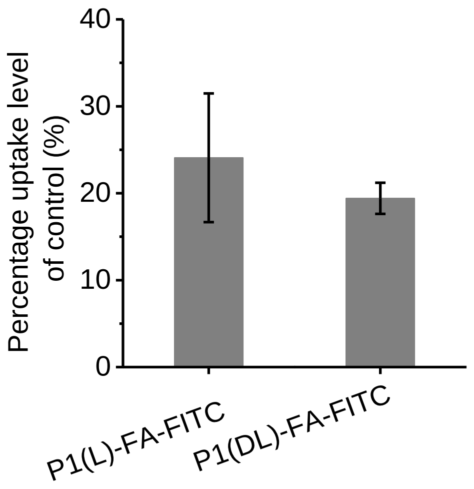

Supplementary Figure 10. Percentage uptake level in HeLa cells at 4 ℃ and pH 7.2. The cellular uptake level at 37 ℃ following 4-h incubation served as 100%. Results represent the means ± s.e.m. of three replicates.

The significantly lower cellular uptake level at 4 ^{o}C verified that both **P1**(L)-FA-FITC and **P1**(DL)-FA-FITC were internalized via folate receptor-mediated endocytocis.

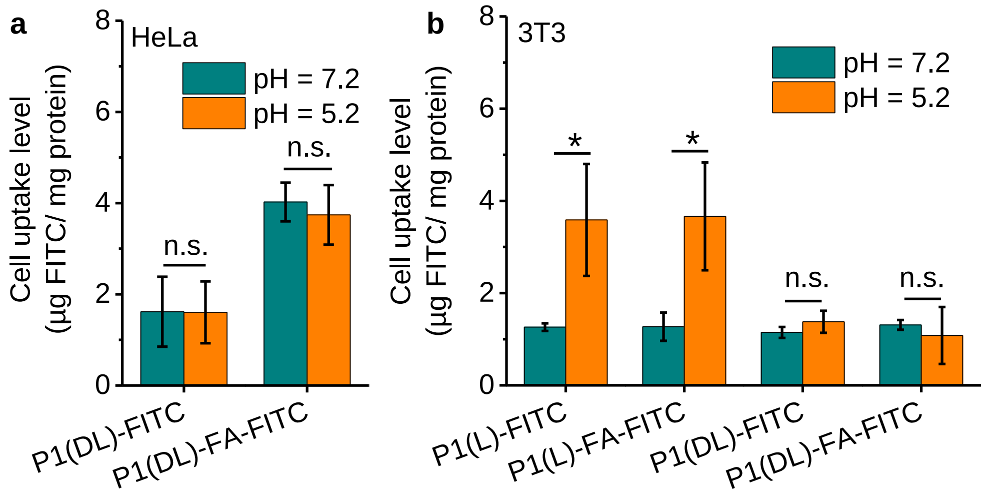

Supplementary Figure 11. Cell uptake results of triazole polypeptides. **a**, Cell uptake of racemic DL-form **P1** in HeLa cells at pH 7.2 and 5.2. **b**, Cell uptake of both L and DL form **P1** in NIH-3T3 cells at pH 7.2 and 5.2. (L-form: coil at pH 7.2 but helical at pH 5.2; DL-form: coil at both pH 7.2 and 5.2). Results represent the means ± s.e.m. of three replicates.

In contrast to the pH-dependent membrane activity of **P1**(L)-FITC and **P1**(L)-FA-FITC (Fig. 5b), the racemic DL-form of **P1** exhibited no differences in uptake level at pH 5.2 and pH 7.2, which supports the conformation-associated membrane activity and excludes the possibility of pH enhanced cell membrane permeability. **P1**(DL)-FA-FITC, however, showed higher uptake level than **P1**(DL)-FITC because of the folate receptor (FR)-mediated endocytosis.

FA-modified triazole polypeptides, **P1**(L)-FA-FITC and **P1**(DL)-FA-FITC, exhibited similar uptake level compared with non-FA analogues in FR-negative NIH-3T3 cells, suggesting cancer cell-specific cell penetration.

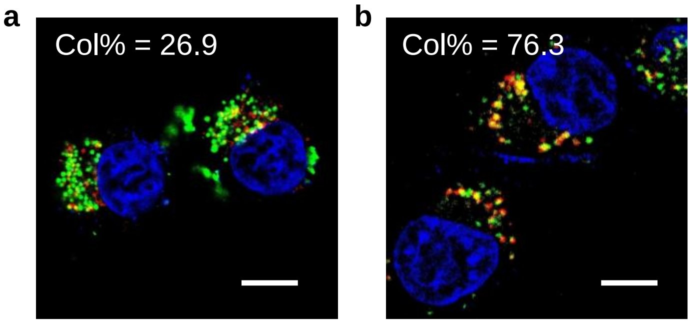

Supplementary Figure 12. Merged CLSM images of HeLa cells following incubation with **P1**(L)-FA-FITC (**a**) or **P1**(DL)-FA-FITC (**b**) at 37 ℃ for 8 h. Cell nuclei were stained with Hoechst 33258 (blue) and endosomes/lysosomes were stained with Lysotracker Red (red). Scale bar represents 15 μm. Col% represents the colocalization ratio of **P1**(L)-FA-FITC or **P1**(DL)-FA-FITC (green) with Lysotracker Red (red) (n = 50).

The CLSM images of HeLa cells with even longer incubation time of **P1**(L)-FA-FITC (8 h, compared with Figure 5d for 4 h) revealed large quantities of green dots fused together and distributed to large areas in the cytoplasm, and some of them appeared permeation patterns as expected, which indicated the effective escape of **P1**(L)-FA-FITC. In consistence with such finding, the colocalization ratio between **P1**(L)-FA-FITC and Lysotracker-Red-stained endosomes further decreased to 26.9%.

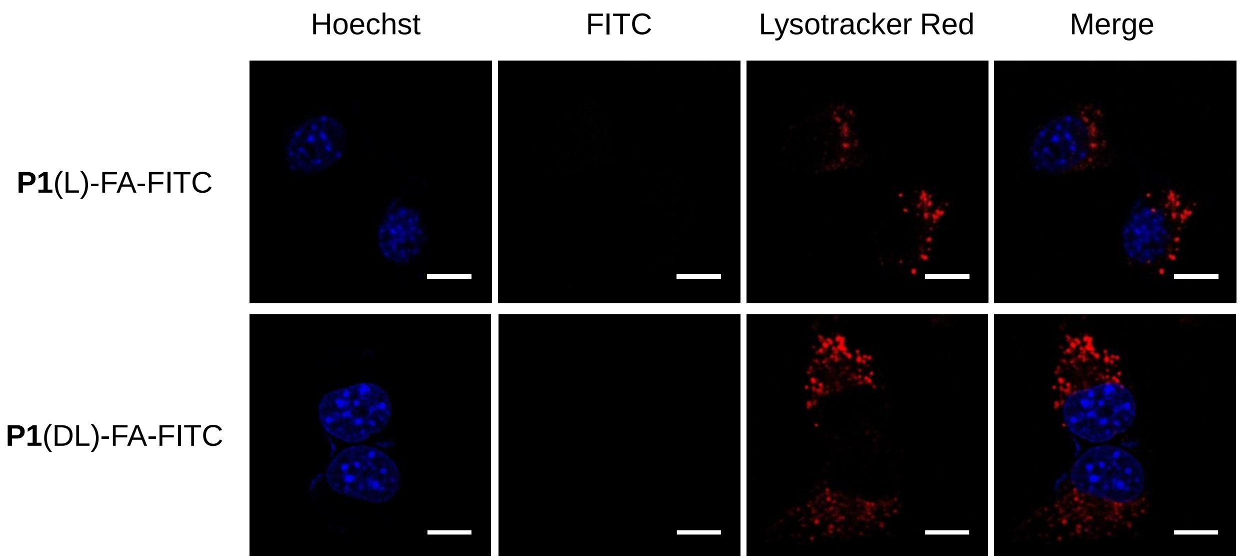

Supplementary Figure 13. CLSM images of 3T3 cells following incubation with **P1**(L)-FA-FITC or **P1**(DL)-FA-FITC at 37 ^{o}C for 4 h. Cell nuclei were stained with Hoechst 33258 and endosomes/lysosomes were stained with Lysotracker Red. Scale bar = 10 μm.

Since the uptake levels of FA-modified triazole polypeptides are low in FR-negative NIH-3T3 cells (as shown in Supplementary Fig. 10), the green fluorescence from fluorescein-labeled polypeptides is weak from CLSM images.

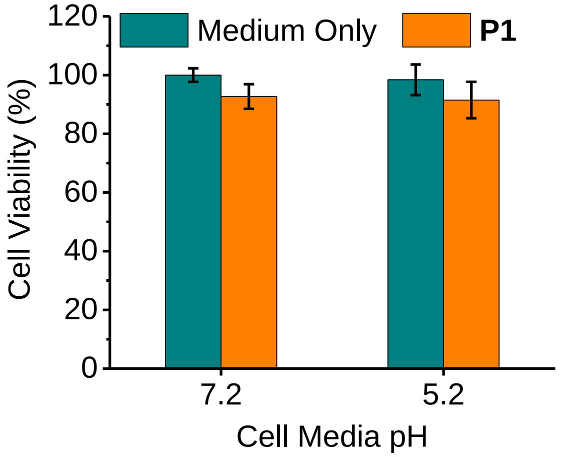

Supplementary Figure 14. Cytotoxicity of **P1** at pH 7.2 and 5.2 by MTT assay. Results represent the means ± s.e.m. of three replicates.

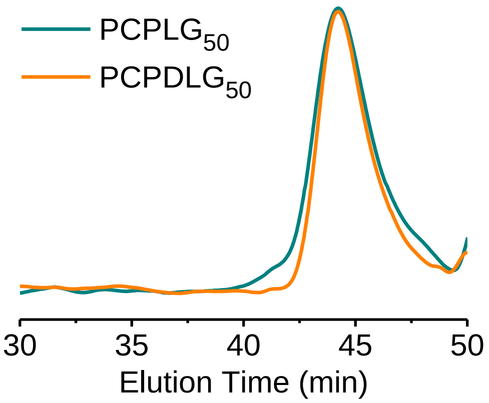

Supplementary Figure 15. GPC traces of $PCPLG_{50}$ and $PCPDLG_{50}$.

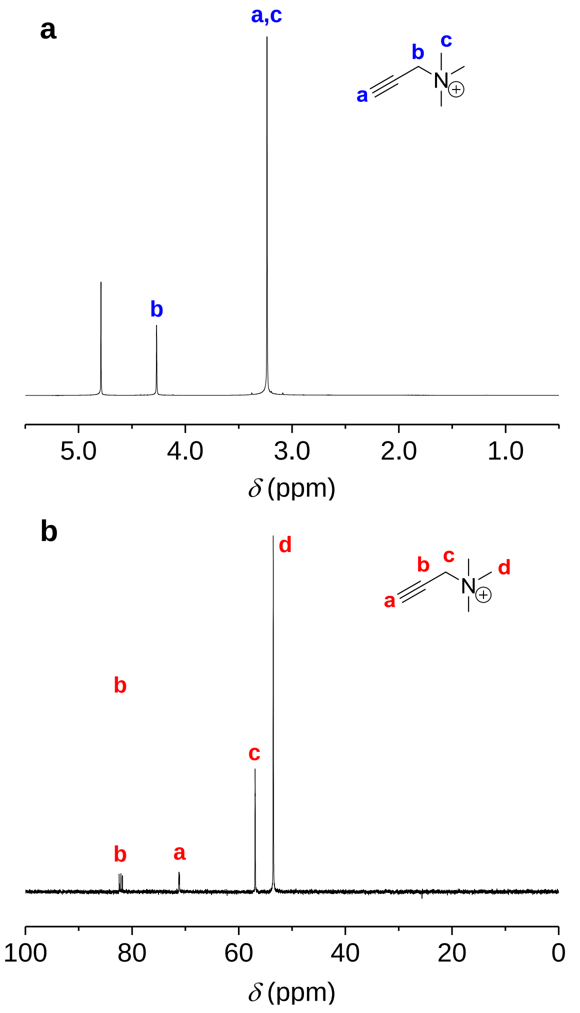

Supplementary Figure 16. ^{1}H (**a**) and ^{13}C (**b**) NMR spectra of PrTA in D_2O.

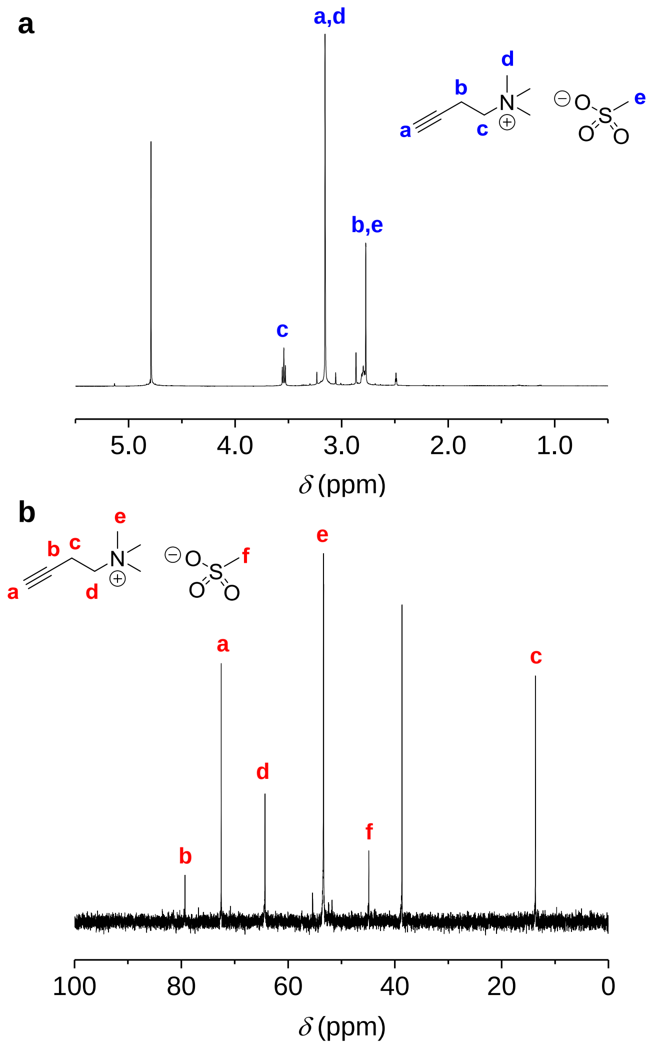

Supplementary Figure 17. ^{1}H (**a**) and ^{13}C (**b**) NMR spectra of BuTA in D_2O.

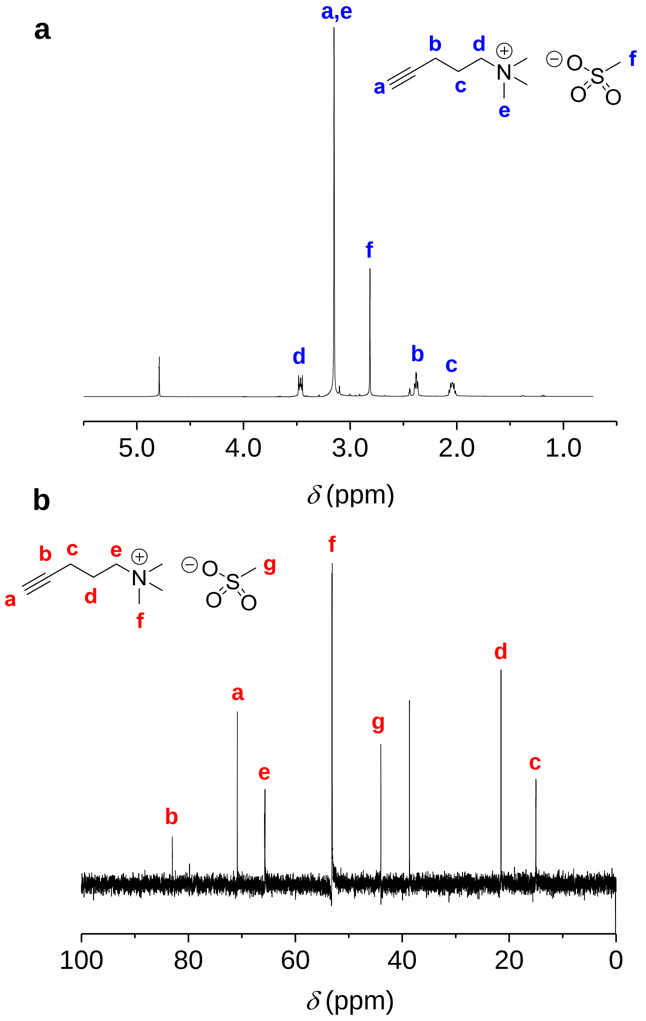

Supplementary Figure 18. ^{1}H (**a**) and ^{13}C (**b**) NMR spectra of PeTA in D_2O.

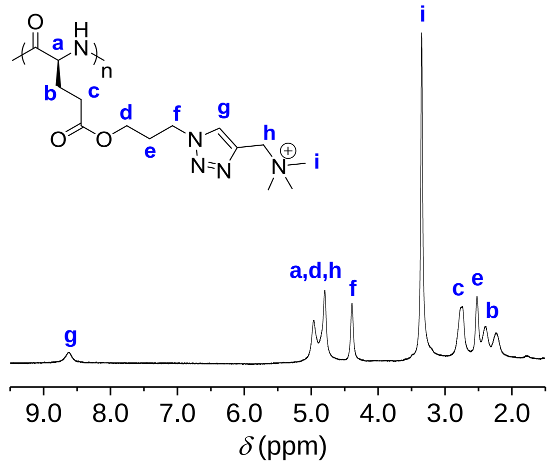

Supplementary Figure 19. ^{1}H NMR spectrum of **P1** in TFA-d.

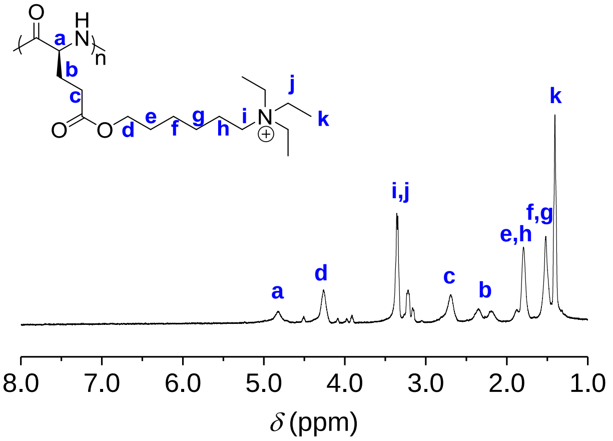

Supplementary Figure 20. ^{1}H NMR spectrum of **P2** in TFA-d.

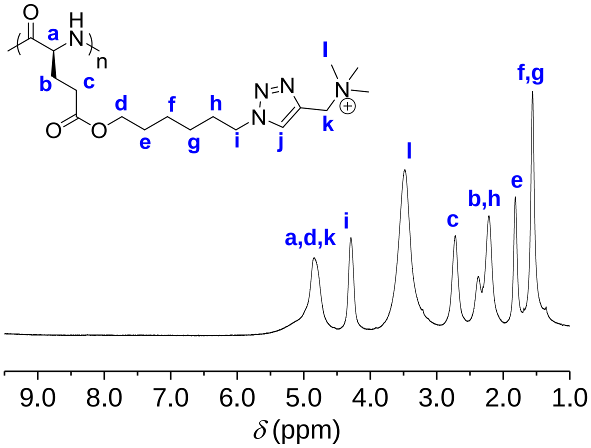

Supplementary Figure 21. ^{1}H NMR spectrum of **P3** in TFA-d.

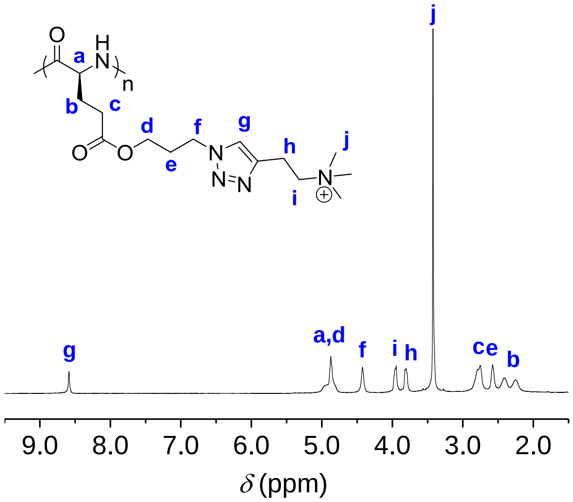

Supplementary Figure 22. ^{1}H NMR spectrum of **P4** in TFA-d.

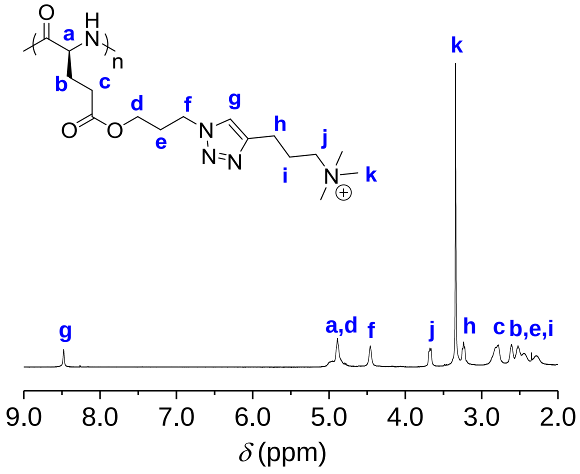

Supplementary Figure 23. ^{1}H NMR spectrum of **P5** in TFA-d.

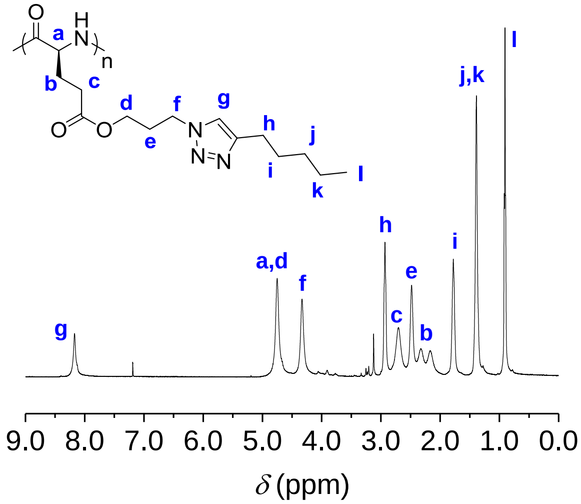

Supplementary Figure 24. ^{1}H NMR spectrum of **P6** in TFA-d.

Supplementary Table 1. Synthesis and characterization of polypeptides bearing chlorine-terminated side chains. [a]

Polymer	M/I [b]	M_n (M_n*) [c] (kDa)	M_w/M_n [d]
$PCPLG_{50}$	50	9.5 [d] (10.3)	1.02
$PCPDLG_{50}$	50	11.3 [d] (10.3)	1.06

[a] Polymerizations were conducted at rt using HMDS as initiator. Monomer conversions were all above 99% monitored by FTIR. [b] Monomer to initiator molar ratio. [c] Obtained molecular weight (theoretical molecular weight). [d] Determined by GPC.

Supplementary Methods

Materials

Anhydrous dimethylformamide (DMF) was dried by a column packed with 4Å molecular sieves and stored in a glovebox. Anhydrous tetrahydrofuran (THF) and hexane were dried by a column packed with alumina. Hexamethyldisilazane (HMDS) was dissolved in anhydrous DMF and stored in the freezer in a glovebox. Spectra/Por RC dialysis tubing with a molecular weight cut-off (MWCO) of 1 kDa was purchased from Spectrum Laboratories (Rancho Dominguez, CA, USA).

Instrumentation

^{1}H NMR spectra were recorded on a Varian U500 spectrometer. Chemical shifts were reported in ppm and referenced to the solvent proton impurities. Gel permeation chromatography (GPC) experiments were performed on a system equipped with an isocratic pump (Model 1100, Agilent Technology, Santa Clara, CA, USA), a DAWN HELEOS multi-angle laser light scattering detector (MALLS) detector (Wyatt Technology, Santa Barbara, CA, USA), and an Optilab rEX refractive index detector (Wyatt Technology, Santa Barbara, CA, USA). The detection wavelength of HELEOS was set at 658 nm. Separations were performed using serially connected size exclusion columns (10^2 Å, 10^3 Å, 10^4 Å, 10^5 Å, and 10^6 Å Phenogel columns, 5 μm, 300 × 7.8 mm, Phenomenex, Torrance, CA, USA) at 60 ℃ using DMF containing 0.1 mol/L LiBr as the mobile phase. The MALLS detector was calibrated using pure toluene and can be used for the determination of the absolute molecular weights (MWs). The MWs of polymers were determined based on the dn/dc value of each polymer sample calculated offline by using the internal calibration system processed by the ASTRA 6 software (version 6.1.1.17, Wyatt Technology, Santa Barbara, CA, USA). MALDI-TOF spectra were collected on a Bruker Ultra Flextreme equipped with a 337 nm nitrogen laser. An accelerating voltage of 25 kV was applied, acquiring 500 shots for each sample. Samples were prepared using α-cyano-4-hydroxycinnamic acid (CHCA) as the matrix (10 mg/mL in THF), and sodium trifluoroacetate as the cationization

agent (10 mg/mL in THF). Samples were dissolved in THF (10 mg/mL). Solutions of matrix, salt, and polymer were mixed in a volume ratio of 4:1:1, respectively. The mixed solutions (0.5 μL) were hand spotted on a stainless steel MALDI target and allowed to dry completely. All spectra were recorded in reflectron mode. Circular dichroism (CD) measurements were carried out on a JASCO J-815 CD spectrometer (JASCO, Easton, MD, USA). The mean residue molar ellipticity of each polypeptide was calculated on the basis of the measured apparent ellipticity by following the literature-reported formulas: Ellipticity ([θ] in deg cm^2 $dmol^{-1}$) = (millidegrees × mean residue weight)/(path length in millimetres × concentration of polypeptide in mg mL^{-1})[1, 2]. Infrared spectra were recorded on a Perkin Elmer 100 serial FTIR spectrophotometer calibrated with polystyrene film (PerkinElmer, Santa Clara, CA, USA). Lyophilization was performed on a FreeZone lyophilizer (Labconco, Kansas City, MO, USA).

Simulation methods

Polypeptide chains were denatured into random coils by applying an artificial stretching potential ("computational tweezers") to separate the terminal Cα atoms and produce an unstructured, elongated configuration. The polypeptides were then placed in a 5×5×5 nm cubic box with periodic boundary conditions and solvated by SPC water molecules[3] to a density of 0.991 g/cm^3 along with 10 or 20 Cl^- ions to maintain charge neutrality. The simulation box was sufficiently large such that with a real space cutoff of 1.0 nm the polypeptide did not interact with its images through the periodic boundary conditions. Molecular dynamics simulations were conducted using the GROMACS 4.6 simulation suite[4]. High energy overlaps in the initial solvated polypeptide configurations were eliminated by steepest descent energy minimization to remove forces exceeding 500 kJ/mol-nm. Simulations were conducted in the NPT ensemble at 298 K and 1 bar employing a Nosé-Hoover thermostat[5] and Parrinello-Rahman barostat[6]. Atoms were randomly assigned initial velocities from a Maxwell distribution at 298 K. Equations of motion were numerically integrated using a leap-frog algorithm with a 2 fs time step[7]. Bond lengths were fixed by the LINCS algorithm to improve efficiency[8]. Coulombic interactions were evaluated using the Particle Mesh Ewald (PME) algorithm with a real-space cutoff of 1.0 nm and

a 0.12 nm Fourier grid spacing[9]. Lennard-Jones interactions were shifted smoothly to zero at 1.0 nm, and Lorentz-Berthelot combining rules used to determine interaction parameters between unlike atoms[10]. A short 1 ns equilibration run was conducted, before commencing a long 100 ns production run using NVIDIA Tesla M2070 GPU cards. Simulation snapshots were harvested for analysis every 50 ps. Simulation trajectories were visualized using VMD[11] and analyzed using the built-in GROMACS tools[4], Matlab R2014a[12], and Matlab R2016a[13].

Structural analyses of each polypeptide were restricted to the terminal 50 ns of the trajectory after the secondary structure had equilibrated. A hydrogen bond is defined as a donor and acceptor atom within 0.35 nm and the H atom inclined by no more than 60° [14, 15]. For the radial distribution function ($g(r_{COM\text{-}OW})$) presented (Fig. 4b), $g(r_{COM\text{-}OW})$ converges to slightly above unity due to the excluded volume of the polypeptide reducing the volume available to the solvent which, for small simulation boxes, imposes a non-negligible reduction in the mean water density computed without accounting for this excluded volume. A finite value of $g(r_{COM\text{-}OW} = 0 \text{ nm})$ is due to the center of mass of the unstructured **P1**-triazole becoming coincident with interstitial solvent molecules residing within the core of the random coil.

Sample preparation and SANS tests

Three D_2O/H_2O solvents with various D_2O percentage (100%, 75%, and 50%, v/v) were used to dissolve the polypeptide sample in order to yield three contrasts in order to probe the water content in the polypeptides. The pH of those solutions were adjusted by adding HCl or NaOH to reach pH = 2.0 or 8.0. The final concentration of the polypeptide is ~ 0.83% (w/v) and the salt concentration is ~ 0.018 M.

SANS experiments were conducted at NGB30mSANS located at the National Institute of Science and Technology (NIST) Center for Neutron Research (NCNR, Gaithersburg, MD, USA). The SANS data were collected at two different sample-to-detector distances (*i.e.* 7 and 4 m) using 6 Å wavelength neutrons, yielding a q range from 0.006 to 0.32 Å^{-1}. Here, q is defined as the scattering vector that $q \equiv \frac{4\pi}{\lambda} sin\frac{\theta}{2}$, where θ is the scattering angle and λ is the neutron wavelength. The 2D raw data were corrected for detector sensitivity, background, sample

transmission, empty cell scattering, and transmission. The corrected data were then circularly averaged, yielding the customary 1D profiles, which were then put on an absolute intensity scale using the measured incident beam flux.

SANS data analysis

SASView 4.0.1 was used to analyze the 1-D scattering patterns[16]. In this study, the scattering intensity, I(q) is expressed as $\phi(SLD_{h\text{-}pep} - SLD_{sol})^2P(q)S(q) + Aq^{-\alpha}$, where ϕ is the volume fraction of the hydrated peptide, $SLD_{h\text{-}pep}$ and SLD_{sol} are the scattering length densities (SLDs) of the hydrated peptide and solvent, P(q) and S(q) are the form and structure factors, corresponding to the intra- and inter-particle interferences, respectively. The multiplication of P(q) and S(q) is used to describe the scattering data of individual polypeptide. Here, we chose a cylinder form factor[17] and a Hayter-Penfold structure factor accounting for interparticle interaction among charged particles[18, 19] based on the enhanced rigidity of the backbone due to the bulky side groups. The second term of the intensity function, $Aq^{-\alpha}$ describes the low-q (from 0.006 to 0.02 $Å^{-1}$) intensity decay observed in the data, implying the large-scale aggregates in the system, whose scattering signal was considered to be independent from that of the individual peptide. Simultaneous fitting was applied to the SANS data of the peptide in acidic and basic conditions, respectively, each with three contrast variations. The global fitting parameters (*i.e.*, the values of the parameters being shared across the samples with different contrast conditions) include the radius and length of cylinder (parameters from P(q)), all parameters in S(q), prefactor A and the power index α. The only local fitting parameter allowed to vary for individual SANS data set was $SLD_{h\text{-}pep}$. The SLD_{sol} was calculated based on the compositions of D_2O/H_2O and kept unvaried during the fitting procedure. The value of ϕ was known from experiment and set to be constant.

Water content analysis

The $SLD_{h\text{-}pep}$ can be expressed below.

$$SLD_{h\text{-}pep} = \varphi_{sol} \times SLD_{sol} + (1 - \varphi_{sol}) \times SLD_{pep} \tag{1}$$

Where $SLD_{h\text{-}pep}$ is the best fitted SLD of the hydrated polypeptide, φ_{sol} is the volume fraction of solvent in the cylinder, SLD_{sol} is the SLD of solvent, and SLD_{pep} is the calculated SLD of the dry polypeptide. Therefore, a linear relationship is expected as $SLD_{h\text{-}pep}$ is plotted versus SLD_{sol} and the slope of the line yields the volume fraction of solvent in the polypeptide. The y-intercept should be the product of the volume fraction of polypeptide, (1 - φ_{sol}) and SLD_{pep} shown as the open symbols in Supplementary Fig. 8. Note that the slight mismatch between the two intercepts in acidic and basic conditions is presumably due to the different volume fraction of solvent. The linearity of data further confirms the best fitting values for $SLD_{h\text{-}pep}$ and φ_{sol} are reasonable.

Synthesis of polypeptide with azide-terminated side chains

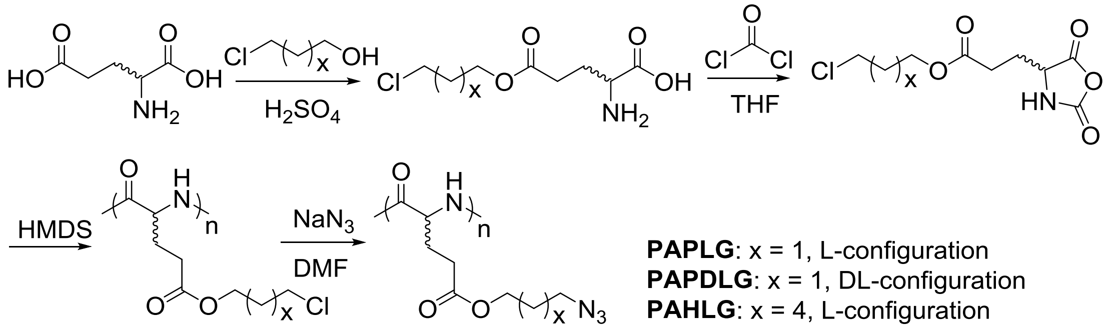

Supplementary Figure 25. Synthetic route to azide-functionalized polypeptides.

Polypeptides with azide-terminated side chains were synthesized following the literature procedure starting from chlorine-funcationalized glutamate (Supplementary Fig. 25)[20]. PAPLG (x = 1, L-configuration) was obtained starting from L-glutamic acid and 3-chloropropanol, PAPDLG (x = 1, DL-configuration) was obtained starting from racemic DL-glutamic acid and 3-chloropropanol, and PAHLG (x = 3, L-configuration) was obtained starting from L-glutamic acid and 6-chlorohexanol.

Synthesis of alkyne-functionalized ammonium salts

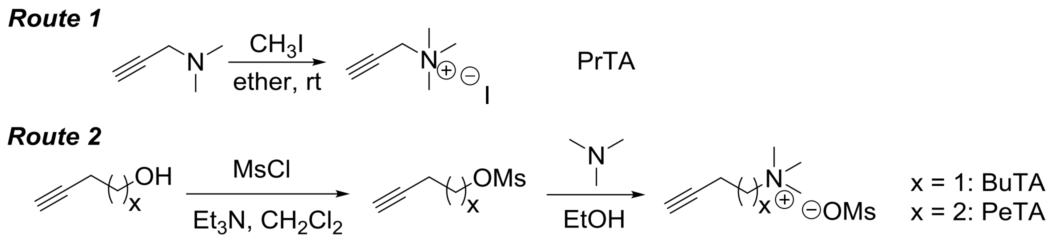

Supplementary Figure 26. Synthetic route to alkyne-functionalized ammonium salts.

Alkyne-functionalized ammonium salts were synthesized through the reaction between electrophiles and tertiary amines. Depending on the spacer lengths between alkyne and ammonium, we developed two routes as shown in Supplementary Fig. 26.

3-Dimethylamino-1-propyne (500 μL, 4.64 mmol) was dissolved in ether (1.0 mL) in a 7-mL glass vial charged with a stir bar, into which ether solution (1.0 mL) of iodomethane (289 μL, 4.64 mmol) was dropwise added. White precipitates were observed immediately. The resulting mixture was stirred at rt overnight. The solid was isolated by centrifugation, washed three times by ether, and dried under vacuum. The product *N*,*N*,*N*-trimethylpropargylammonium iodide (PrTA) was obtained as white powder (913 mg, 87% yield). ^{1}H NMR (D_2O): δ 4.27 (s, 2H, HC≡CCH_2-), 3.24 (s, 10H, $(CH_3)_3N^+$ - and *H*C≡C-). ^{13}C NMR (D_2O): δ 82.1, 71.2, 56.5, 53.0. HR-MS (ESI): *m*/*z* $[M]^+$ Calcd. for $C_6H_{12}N$ 98.0970; Found: 98.0971.

But-3-yn-1-yl methanesulfonate (BuOMs) and pent-4-yn-1-yl methanesulfonate (PeOMs) were synthesized following a literature procedure[21]. Methanesulfonylchloride (6.0 mL, 77.5 mmol) was added dropwise to a stirred solution of 3-butyn-1-ol (4.0 mL, 52.8 mmol) and triethylamine (10.5 mL, 75.3 mmol) in dichloromethane (50 mL) at 0 °C. After stirring at rt overnight, water (100 mL) was added to the reaction mixture. The organic layer was separated, washed with water (100 mL × 4), and dried over anhydrous sodium sulfate. The final product BuOMs was obtained as a brown oily liquid after the removal of solvent (9.8 g, 90% yield). ^{1}H NMR ($CDCl_3$): δ 4.30 (t, *J* = 6.7 Hz, 2H, $CH_3SO_3CH_2$-), 3.05 (s, 3H, CH_3SO_2-), 2.66 (dt, *J* = 6.7, 2.6 Hz, 2H, HC≡CCH_2-), 2.06 (t, *J* = 2.6 Hz, 1H, *H*C≡C-). ^{13}C NMR ($CDCl_3$): δ 78.6, 70.9, 67.0,

37.6, 19.7.

PeOMs was synthesized with the same method using 4-pentyn-1-ol (93% yield). ^{1}H NMR ($CDCl_3$): δ 4.34 (t, *J* = 6.1 Hz, 2H, $CH_3SO_3CH_2$-), 3.02 (s, 3H, CH_3SO_2-), 2.36 (dt, *J* = 6.8, 2.7 Hz, 2H, HC≡CCH_2-), 2.00 (t, *J* = 2.7 Hz, 1H, *H*C≡C-), 1.95 (m, 2H, HC≡CCH_2CH_2-). ^{13}C NMR ($CDCl_3$): δ 82.0, 69.8, 68.2, 37.2, 27.7, 14.6.

BuOMs (1 g, 6.75 mmol) was dissolved in ethanol in a Schlenk tube, trimethylamine (4.2 M in ethanol, 5.0 mL, 21 mmol) was added and the solution was refluxed at 70 °C overnight. The solvent and excessive trimethylamine were removed under vacuum. The product BuTA was recrystallized in ethanol to yield a white crystal (0.96 g, 69% yield). ^{1}H NMR (D_2O): δ 3.40 (t, *J* = 7.1 Hz, 2H, $(CH_3)_3N^+CH_2$-), 3.01 (s, 10H, $(CH_3)_3N^+CH_2$- and *H*C≡C-), 2.66 (m, 2H, HC≡CCH_2-), 2.63 (s, 3H, $CH_3SO_3^-$). ^{13}C NMR (D_2O): δ 79.2, 72.5, 64.3, 53.3, 44.8, 13.6. HR-MS (ESI): *m*/*z* $[M]^+$ Calcd. for $C_7H_{14}N$ 112.1126; Found: 112.1128.

PeTA was synthesized with the same method using PeOMs (73% yield). ^{1}H NMR (D_2O): δ 3.34 (m, 2H, $(CH_3)_3N^+CH_2$-), 3.03 (s, 10H, $(CH_3)_3N^+CH_2$- and *H*C≡C-), 2.69 (s, 3H, $CH_3SO_3^-$), 2.26 (dt, *J* = 6.7, 2.8 Hz, 2H, HC≡CCH_2-), 1.92 (m, 2H, HC≡CCH_2CH_2-). ^{13}C NMR (D_2O): δ 83.0, 70.9, 65.7, 53.1, 44.0, 21.5, 15.0. HR-MS (ESI): *m*/*z* $[M]^+$ Calcd. for $C_8H_{16}N$ 126.1283; Found: 126.1285.

Synthesis of triazole polypeptides

P1, P3-P6 were synthesized with copper-catalyzed Huisgen click chemistry following a literature procedure[20, 22] (Supplementary Fig. 27).

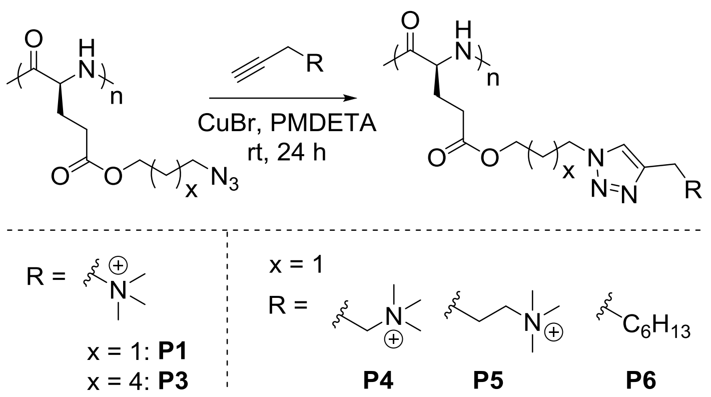

Supplementary Figure 27. Synthetic route to triazole polypeptides.

In a glovebox, PAPLG (40 mg, 0.19 mmol of side-chain azido groups), PMDETA (19.7 μL, 0.09 mmol) and alkyne-functionalized small molecules (0.28 mmol) were dissolved in DMF (1 mL). The resulting solution was transferred into a small vial charged with CuBr (2.7 mg, 0.02 mmol) and a magnetic stir bar. The mixture was stirred at room temperature for 24 h. The reaction was then quenched by transferring out of glovebox and exposing to air. The solution was first dialyzed against EDTA/NaCl aqueous solution for 1 h to promote copper removal and anion exchange (MWCO = 1 kDa), and then further purified by dialysis against DI water for 6 h (DI water changed every hour). The final triazole polypeptides were obtained as light yellow powder (65-82% yield).

P1 was synthesized with the reaction between PAPLG and PrTA. ^{1}H NMR (TFA-d): δ 8.52 (s, 1H, Triazole-H), 4.96-4.60 (m, 5H, α-H, -COOCH_2CH$_2$CH$_2$-, and -CH_2N$^+$(CH$_3$)$_3$), 4.28 (s, 2H, -COOCH$_2$CH$_2$CH_2-), 3.24 (s, 9H, (CH_3)$_3$N$^+$-), 2.63 (s, 2H, -CH$_2$CH_2COO-), 2.42 (s, 2H, -COOCH$_2$CH_2CH$_2$-), 2.35-2.03 (m, 2H, -CH_2CH$_2$COO-).

P3 was synthesized with the reaction between PAHLG and PrTA. ^{1}H NMR (TFA-d): δ 4.85 (s, 5H, α-H, -CH_2(CH$_2$)$_4$CH$_2$OOC- and (CH_3)$_3$N$^+$CH_2-), 4.29 (s, 2H, -CH_2OOC-), 3.47 (s, 9H, (CH_3)$_3$N$^+$-), 2.72 (s, 2H, -CH$_2$CH_2COO-), 2.48-2.06 (m, 4H, -CH_2(CH$_2$)$_3$CH$_2$OOC- and -CH_2CH$_2$COO-), 1.82 (s, 2H, -CH_2CH$_2$OOC-),), 1.56 (s, 4H, -(CH_2)$_2$CH$_2$CH$_2$OOC-).

P4 was synthesized with the reaction between PAPLG and BuTA. ^{1}H NMR (TFA-d): δ 8.46

(s, 1H, Triazole-H), 4.91-4.64 (m, 3H, α-H and -COOCH_2CH$_2$CH$_2$-), 4.30 (s, 2H, -COOCH$_2$CH$_2$CH_2-), 3.83 (m, 2H, -CH$_2$CH_2N$^+$(CH$_3$)$_3$), 3.69 (m, 2H, -CH_2CH$_2$N$^+$(CH$_3$)$_3$), 3.30 (s, 9H, (CH_3)$_3$N$^+$-), 2.63 (s, 2H, -CH$_2$CH_2COO-), 2.45 (s, 2H, -COOCH$_2$CH_2CH$_2$-), 2.37-2.06 (m, 2H, -CH_2CH$_2$COO-).

P5 was synthesized with the reaction between PAPLG and PeTA. ^{1}H NMR (TFA-d): δ 8.37 (s, 1H, Triazole-H), 4.94-4.66 (m, 3H, α-H and -COOCH_2CH$_2$CH$_2$-), 4.35 (s, 2H, -COOCH$_2$CH$_2$CH_2-), 3.56 (m, 2H, -CH$_2$CH$_2$CH_2N$^+$(CH$_3$)$_3$), 3.23 (s, 9H, (CH_3)$_3$N$^+$-), 3.13 (m, 2H, -CH_2CH$_2$CH$_2$N$^+$(CH$_3$)$_3$), 2.67 (s, 2H, -CH$_2$CH_2COO-), 2.50 (s, 2H, -COOCH$_2$CH_2CH$_2$-), 2.46-2.27 (m, 2H, -CH_2CH$_2$COO-), 2.17 (s, 2H, -CH$_2$CH_2CH$_2$N$^+$(CH$_3$)$_3$).

P6 was synthesized with the reaction between PAPLG and 1-heptyne. Since **P6** is not water soluble, we use precipitation to purify **P6** instead of dialysis. After the quenching of Cu(I) with air, the copper salts were filtered through a packed neutral Al_2O_3 column. **P6** was then purified by precipitation with hexane/ether three times (1:1, v/v). ^{1}H NMR (TFA-d): δ 8.17 (s, 1H, Triazole-H), 4.75 (s, 3H, α-H and -COOCH_2CH$_2$CH$_2$-), 4.34 (s, 2H, -COOCH$_2$CH$_2$CH_2-), 2.93 (s, 2H, -CH_2CH$_2$(CH$_2$)$_2$CH$_3$), 2.70 (s, 2H, -CH$_2$CH_2COO-), 2.48 (s, 2H, -COOCH$_2$CH_2CH$_2$-), 2.40-2.09 (m, 2H, -CH_2CH$_2$COO-), 1.78 (s, 2H, -CH$_2$CH_2(CH$_2$)$_2$CH$_3$), 1.38 (s, 4H, -CH$_2$CH$_2$(CH_2)$_2$CH$_3$), 0.91 (m, 3H, J = 6.5 Hz, -CH$_2$CH$_2$(CH$_2$)$_2$CH_3),.

For the fluorescein-labelled and folic acid (FA)-modified **P1**, the corresponding small molecular alkynes were first synthesized following the literature procedures. Propargyl fluorescein was synthesized by reacting propargyl amine with fluorescein isothiocyanate (FITC) in DMF[23]; while propargyl folate was synthesized by reacting propargyl amine with the NHS ester of FA in DMSO[24]. The resulting propargyl fluorescein and propargyl folate was then mixed with PrTA with different molar ratios, and co-clicked with PAPLG or PAPDLG in the glovebox. The feeding molar percentage of propargyl fluorescein was fixed at 2.5 mol%. The resulting four polymers were named as **P1**(L)-FITC (starting with PAPLG, no FA incorporation), **P1**(L)-FA-FITC (starting with PAPLG, 10 mol% FA incorporation), **P1**(DL)-FITC (starting with PAPDLG, no FA incorporation), **P1**(DL)-FA-FITC (starting with PAPDLG, 10 mol% FA

incorporation). The chemical structures of these four polypeptides are shown in Supplementary Fig. 9a.

Synthesis of control polypeptides without side-chain triazoles

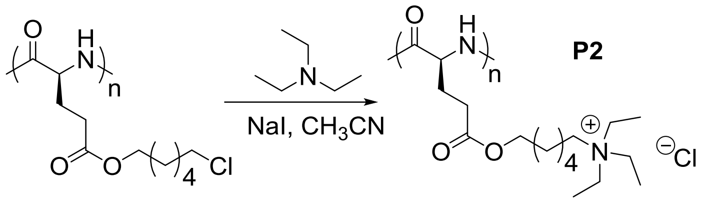

Supplementary Figure 28. Synthetic route to control polypeptides without side-chain triazoles.

P2 were synthesized through nucleophilic reaction of side-chain terminal chlorine with tertiary amine[25]. We first tried to synthesize trimethylammonium based polypeptides for good comparison with **P1**, however, the reaction failed since the high reactivity of starting material trimethylamine caused serious side-reactions. We therefore use triethylamine instead as the nucleophile. Typically, poly(γ-(6-chlorohexyl)-L-glutamate) (PCHLG, 86.5 mg, 0.35 mmol of side-chain chloro groups) was dissolved in DMF (2.0 mL), NaI (157 mg, 1.05 mmol) was dissolved in acetonitrile (2.0 mL). Both solutions were transferred to a 25-mL Schlenk tube, into which triethylamine (97.5 μL, 0.70 mmol) was added. The resulting mixture was then stirred at 80 °C for 48 h. After most DMF and acetonitrile was removed under vacuum, NaCl aqueous solution (1.0 M, 3 mL) was added and the solution stirred for 3 h at rt to promote anion exchange. The product was purified by dialysis (MWCO = 1 kDa) against DI water for 1 day (DI water change every 3 h). The product **P2** was obtained as light yellow powder after lyophilization (88.2 mg, 72% yield). ^{1}H NMR (TFA-d): δ 4.71 (s, 1H, α-H), 4.15 (s, 2H, -COOCH_2(CH_2)$_4$$CH_2$-), 3.30-3.01 (m, 8H, -C$H_2$$N^+$(C$H_2$$CH_3$)$_3$), 2.58 (s, 2H, -$CH_2CH_2$COO-), 2.31-2.01 (m, 2H, -C$H_2$$CH_2$COO-), 1.75-1.35 (m, 8H, -COOCH_2(CH_2)$_4$$CH_2$-), 1.28 (s, 9H, (C$H_3$$CH_2$)$_3$$N^+$-).

Supplementary References

1. Adler, A. J., Greenfield, N. J., Fasman, G. D. Circular dichroism and optical rotatory dispersion of proteins and polypeptides. In: C. H. W. Hirs SNT (ed). *Methods in Enzymology* vol. 27. (Academic Press, 1973), 675-735.
2. Greenfield, N. J. Using circular dichroism spectra to estimate protein secondary structure. *Nat. Protoc.* **1,** 2876-2890 (2006).
3. Berendsen, H. J. C., Postma, J. P. M., van Gunsteren, W. F., Hermans, J. Interaction models for water in relation to protein hydration. In: Pullman B (ed). *Intermolecular Forces*. (Springer Netherlands, Dordrecht, 1981), 331-342.
4. Hess, B., Kutzner, C., van der Spoel, D., Lindahl, E. GROMACS 4: algorithms for highly efficient, load-balanced, and scalable molecular simulation. *J. Chem. Theory Comput.* **4,** 435-447 (2008).
5. Nosé, S. A unified formulation of the constant temperature molecular dynamics methods. *J. Chem. Phys.* **81,** 511-519 (1984).
6. Parrinello, M., Rahman, A. Polymorphic transitions in single crystals: a new molecular dynamics method. *J. Appl. Phys.* **52,** 7182-7190 (1981).
7. Hockney, R. W., Eastwood, J. W. *Computer Simulation Using Particles* (CRC Press, Bristol, 2010).
8. Hess, B., Bekker, H., Berendsen, H. J. C., Fraaije, J. G. E. M. LINCS: A linear constraint solver for molecular simulations. *J. Comput. Chem.* **18,** 1463-1472 (1997).
9. Essmann, U., *et al*. A smooth particle mesh Ewald method. *J. Chem. Phys.* **103,** 8577-8593 (1995).
10. Allen, M. P., Tildesley, D. J. *Computer Simulation of Liquids* (Oxford University Press, Oxford, 1989).
11. Humphrey, W., Dalke, A., Schulten, K. VMD: visual molecular dynamics. *J. Mol. Graphics* **14,** 33-38 (1996).
12. MATLAB 2014a, The MathWorks, Inc., Natick, Massachusetts, United States.
13. MATLAB 2016a, The MathWorks, Inc., Natick, Massachusetts, United States.
14. Kumar, R., Schmidt, J. R., Skinner, J. L. Hydrogen bonding definitions and dynamics in liquid water. *J. Chem. Phys.* **126,** 204107 (2007).
15. Baker, E. N., Hubbard, R. E. Hydrogen bonding in globular proteins. *Prog. Biophys. Mol. Biol.* **44,** 97-179 (1984).
16. SasView, http://www.sasview.org/.
17. Guinier, A., Fournet, G. *Small-Angle Scattering of X-Rays* (John Wiley and Sons, New York, 1955).
18. Hayter, J. B., Penfold, J. An analytic structure factor for macroion solutions. *Mol. Phys.* **42,** 109-118 (1981).
19. Hansen, J. P., Hayter, J. B. A rescaled MSA structure factor for dilute charged colloidal dispersions. *Mol. Phys.* **46,** 651-656 (1982).
20. Tang, H., Yin, L., Kim, K. H., Cheng, J. Helical poly(arginine) mimics with superior

cell-penetrating and molecular transporting properties. *Chem. Sci.* **4,** 3839-3844 (2013).
21. Saito, Y., *et al.* C8-alkynyl- and alkylamino substituted 2'-deoxyguanosines: a universal linker for nucleic acids modification. *Tetrahedron* **64,** 3578-3588 (2008).
22. Tang, H., Zhang, D. General route toward side-chain-functionalized α-helical polypeptides. *Biomacromolecules* **11,** 1585-1592 (2010).
23. Xu, C., Ye, L. Clickable molecularly imprinted nanoparticles. *Chem. Commun.* **47,** 6096-6098 (2011).
24. De, P., Gondi, S. R., Sumerlin, B. S. Folate-conjugated thermoresponsive block copolymers: highly efficient conjugation and solution self-assembly. *Biomacromolecules* **9,** 1064-1070 (2008).
25. Song, Z., *et al.* Polypeptides with quaternary phosphonium side chains: synthesis, characterization, and cell-penetrating properties. *Biomacromolecules* **15,** 1491-1497 (2014).
26. Metcalfe, J. C. Nuclear Magnetic Resonance Spectroscopy. *Physical Principles and Techniques of Protein Chemistry*. (Academic Press, New York, 1970), 275-363.
